# Comparative clinical, virological and pathological characterization of equine rotavirus A G3P[12] and G14P[12] infection in neonatal mice

**DOI:** 10.1099/jgv.0.002110

**Published:** 2025-06-05

**Authors:** Chandika Gamage, William Holl, Viviana Parreño, Côme J. Thieulent, Udeni B. R. Balasuriya, M. Aldana Vissani, Maria E. Barrandeguy, Mariano Carossino

**Affiliations:** 1 Department of Pathobiological Sciences, School of Veterinary Medicine, Louisiana State University, Baton Rouge, LA 70803, USA; 2 Louisiana Animal Disease Diagnostic Laboratory, School of Veterinary Medicine, Louisiana State University, Baton Rouge, LA 70803, USA; 3 Instituto de Virología, CICVyA, Instituto Nacional de Tecnología Agropecuaria (INTA), Buenos Aires B1686, Argentina; 4 Consejo Nacional de Investigaciones Científicas y Técnicas (CONICET), Buenos Aires C1425, Argentina; 5 Escuela de Veterinaria, Universidad del Salvador, Buenos Aires B1630, Argentina; 6 Plataforma de Salud Animal, Instituto Nacional de Investigación Agropecuaria (INIA), La Estanzuela, Semillero, Uruguay

**Keywords:** bovine rotavirus A, equine rotavirus A, G3P[12], G14P[12], neonatal mice, porcine rotavirus A, rotavirus A, rotavirus tropism

## Abstract

Group A rotavirus (RVA) infections are a leading cause of neonatal diarrhoea in foals. Neonatal mice could serve as a useful tool to study the pathogenesis of equine RVA (ERVA) as well as a preclinical model for assessment of vaccine efficacy. This study aimed to comparatively evaluate the clinical, virological and pathological features of ERVA G3P[12] and G14P[12] infection in neonatal mice and compare them with porcine OSU G5P[7] and bovine UK G6P[5] RVA reference strains. Neonatal mice orally inoculated with equine, bovine and porcine RVA developed short-lived diarrhoea at variable rates, G14P[12] (61%) and G3P[12] (88%). Viral replication kinetics for all strains were characterized by a gradual decline in viral load to levels below the limit of detection by 72–96 h post-infection (hpi), in line with the reduction in the number of infected enterocytes demonstrated via RNAscope^®^ *in situ* hybridization. Importantly, the clinical and viral replication kinetics correlated with significant microscopic intestinal alterations characterized by enterocyte vacuolation, scalloping and hyperplasia with a peak occurring at 48 hpi and persisting until at least 96 hpi. Overall, neonatal mice develop a disease phenotype of short duration following infection with equine, porcine and bovine RVA strains characterized by diarrhoea and pronounced histological alterations in the intestinal villi. The limited intestinal viral replication is likely associated with host restriction. The clinical and pathological phenotypes developed by neonatal mice following experimental infection could serve as a preclinical tool to assess vaccine efficacy and for pathogenesis studies involving RVA of equine, porcine and bovine origin.

## Introduction

Group A rotaviruses (RVA) are non-enveloped RNA viruses with a triple capsid and a segmented genome composed of 11 double-stranded RNA segments that belong to the family *Sedoreoviridae* [1]. Based on their outer capsid proteins VP4 and VP7, RVA strains are classified in P- and G-types, respectively [2]. RVA infections are considered one of the leading causes of gastroenteritis in children and neonatal farm animals, contributing to significant morbidity and mortality worldwide [3]. Despite the availability of vaccines, RVA remains a public health concern, particularly in low-income countries where vaccination coverage is limited and efficacy is hampered by higher rates of RVA diversity [4]. In addition to genotype diversity, several factors contribute to reduced oral rotavirus A vaccine efficacy, especially in low-income settings. These include maternal antibody interference, environmental enteric dysfunction, altered gut microbiota, concurrent enteric infections and host genetic factors [5,8]. RVA infections in livestock (particularly young calves, weaning and post-weaning piglets and foals) are a substantial cause of economic losses due to decreased productivity, increased veterinary costs and mortality [9,11]. Equine RVA (ERVA) is responsible for causing 20–77% of diarrhoea cases in foals under 3 months of age [12,16], leading to severe dehydration and potentially fatal outcomes. The most epidemiologically relevant ERVA strains are those belonging to the genotypes G3P[12] and G14P[12], which widely circulate in the equine population worldwide [17]. Inactivated vaccines are the most common for rotavirus prevention, with commercially available options, including ScourGuard^®^ 4K, ScourGuard^®^ 4KC (Zoetis Animal Health, Kalamazoo, MI, USA), Fencovis^®^ (Boehringer Ingelheim, Ingelheim am Rhein, Germany), Bovilis^®^ Rotavec^®^ and Bovilis^®^ Guardian^®^ (Merck Animal Health, USA) for bovine RVA (BRVA). For porcine RVA (PRVA), there are two live attenuated vaccines licensed in the USA: ProSystem^®^ Rota and ProSystem^®^ RCE (Merck Animal Health). Vaccines available for ERVA are inactivated and include the H2 (G3P[12]) strain-based vaccines by Zoetis Animal Health (USA, New Zealand, Australia, Europe), Rotamix Equin (Biochemiq, Argentina) and HO-5 (G3BP[12]) strain-based vaccines licensed in Japan by Nisseiken (Tokyo, Japan), all administered to pregnant mares to enhance colostral immunity for neonatal protection [18,20]. These antibodies are actively concentrated in colostrum due to the expression of the neonatal Fc receptor in the mammary gland during calostrogenesis. However, the vaccine efficacy is limited, partly due to the high genetic diversity across circulating RVA strains and the difficulties in obtaining field isolates to be included in current vaccine formulations, which limits the potential to broaden protection [21,24].

Rotavirus pathogenesis has been extensively studied [25,26]. This group of enteric viruses infects the intestinal enterocytes at the tip of the intestinal villi, inducing their degeneration and subsequent villous atrophy/blunting, causing short-term non-bloody diarrhoea with minimal inflammation in the small intestine [25,28]. Host–virus interactions at the enterocyte level are primarily explored through surrogate cell cultures and animal models, as human or animal-specific enterocyte models are challenging to establish [29]. Although various animal models, such as piglets and calves, have been employed to study RVA pathogenesis and virus-specific immunity, these models are often limited by ethical considerations, logistical challenges and high maintenance costs [30]. Consequently, there is growing interest in alternative models for studying viral pathogenesis and the efficacy of vaccines and therapeutics. The neonatal (suckling) mouse model was first described in 1963 [31]. This became a valuable tool for studying rotavirus pathogenesis due to its susceptibility to different rotavirus strains, ease of handling and the ability to develop diarrhoea [31,36]. Genetically modified mouse strains allow detailed investigation of host factors affecting RVA pathogenesis and immunity, providing insights into molecular mechanisms of susceptibility and resistance that are challenging to study in other models. Multiple studies have utilized this model to study RVA immunity [29,33], investigate the genetic basis of RVA host range restriction [34], characterize field isolates of animal rotaviruses [23,24, 35] and assess vaccine efficacy. However, knowledge regarding the susceptibility and permissiveness of this murine model to ERVA infection, including viral replication dynamics and induction of intestinal pathology, remains extremely limited [36]. Hence, there is a critical need to comprehensively and comparatively characterize ERVA infection dynamics in the neonatal mouse model to determine its suitability for pathogenesis and vaccine efficacy studies.

In this study, we conducted a time course analysis of suckling mice infected with ERVA G3P[12] and G14P[12] strains and compared the results with those of PRVA G5P[7] and BRVA G6P[5] strains, examining clinical, virological and pathological outcomes. We demonstrated that suckling mice develop a similar disease phenotype with short-lived diarrhoea correlated with pronounced intestinal histological alterations following infection with all ERVA, PRVA and BRVA strains. This study provides a unique comparative characterization of the infection dynamics of different livestock RVA strains, increasing our knowledge of this model and its features that could be of potential use for assessing the efficacy of vaccines and therapeutics against RVA.

## Methods

### Cells and viruses

The African green monkey kidney MA-104 cell line (CRL-2378.1^™^, American Type Culture Collection, Manassas, VA, USA; RRID: CVCL_3845) was maintained in Eagle’s minimum essential medium (MEM, Cat# 10–010-CV, Corning, NY, USA) with l-glutamine and supplemented with 10% heat-inactivated FBS (HyClone^™^, Cytiva, Marlborough, MA, USA), 1 mM sodium pyruvate (Gibco^®^, Waltham, MA, USA), 1X non-essential amino acids, penicillin and streptomycin [100 U ml^−1^ and 100 µg ml^−1^, respectively (Gibco^®^)] and 0.25 µg ml^−1^ of amphotericin B (Gibco^®^). Cells were maintained in a humidified incubator at 37 °C and 5% CO_2_. ERVA strains H2 (G3P[12]) and MCBI (RVA/Horse-tc/ARG/E8701-6MCBI/2016/G14P[12]), PRVA strain OSU G5P[7] (RVA/Pig-tc/USA/1975/OSU/G5P[7]) and BRVA strain UK (G6P[5]) (RVA/Bovine-tc/USA/UK/1984/G6P[5]) were sourced as previously described [17,37] and propagated in MA-104 cells, as indicated below.

### Rotavirus propagation, titration and inoculum preparation

Rotaviruses were propagated in MA-104 cells grown in TripleFlask^®^ (Thermo Scientific, Waltham, MA, USA). Confluent monolayers of MA-104 cells were washed three times with serum-free MEM and inoculated with trypsin-activated (0.5 µg ml^−1^, Cat# T4799, Sigma-Aldrich, St. Louis, MO, USA) rotaviruses at a multiplicity of infection of 0.1 as previously described [17]. The inoculated flasks were incubated at 37 °C in a CO_2_ incubator for 24–72 h until 100% cytopathic effect was observed. Following one freeze-thaw cycle at −80 °C, infected cell lysates were clarified through centrifugation at 670 ***g*** for 10 min at 4 °C, filtered through a 0.45 µm filter and finally centrifuged at 30,000 ***g*** for 3 h to pellet virus particles. The virus pellet was resuspended in 300 µl of sterile Tris NaCl-CaCl_2_ buffer [10 mM Tris (pH 7.4), 140 mM NaCl, 5 mM CaCl_2_], divided into 100 µl aliquots and stored at −80 °C. Virus stocks prepared for experimental infection were titrated using the tissue culture infectious dose 50% (TCID_50_) method, as previously described [17], and end-point viral titers were calculated using the Reed–Muench method [38]. On the day of experimental infection, inocula containing rotavirus suspensions at 1×10^7^ TCID_50_ in 100 µl of MEM with 5% (w/v) sodium bicarbonate (Cat# S5761-500G, Sigma-Aldrich) were prepared.

### Mice

Six- to eight-week-old timed-pregnant BALB/c female mice on the 16th day of gestation (E16) were purchased from Charles River Laboratories (Wilmington, MA, USA) and maintained in an animal biosafety level 2 facility.

### Experimental design and oral inoculation

**Fig. 1.**
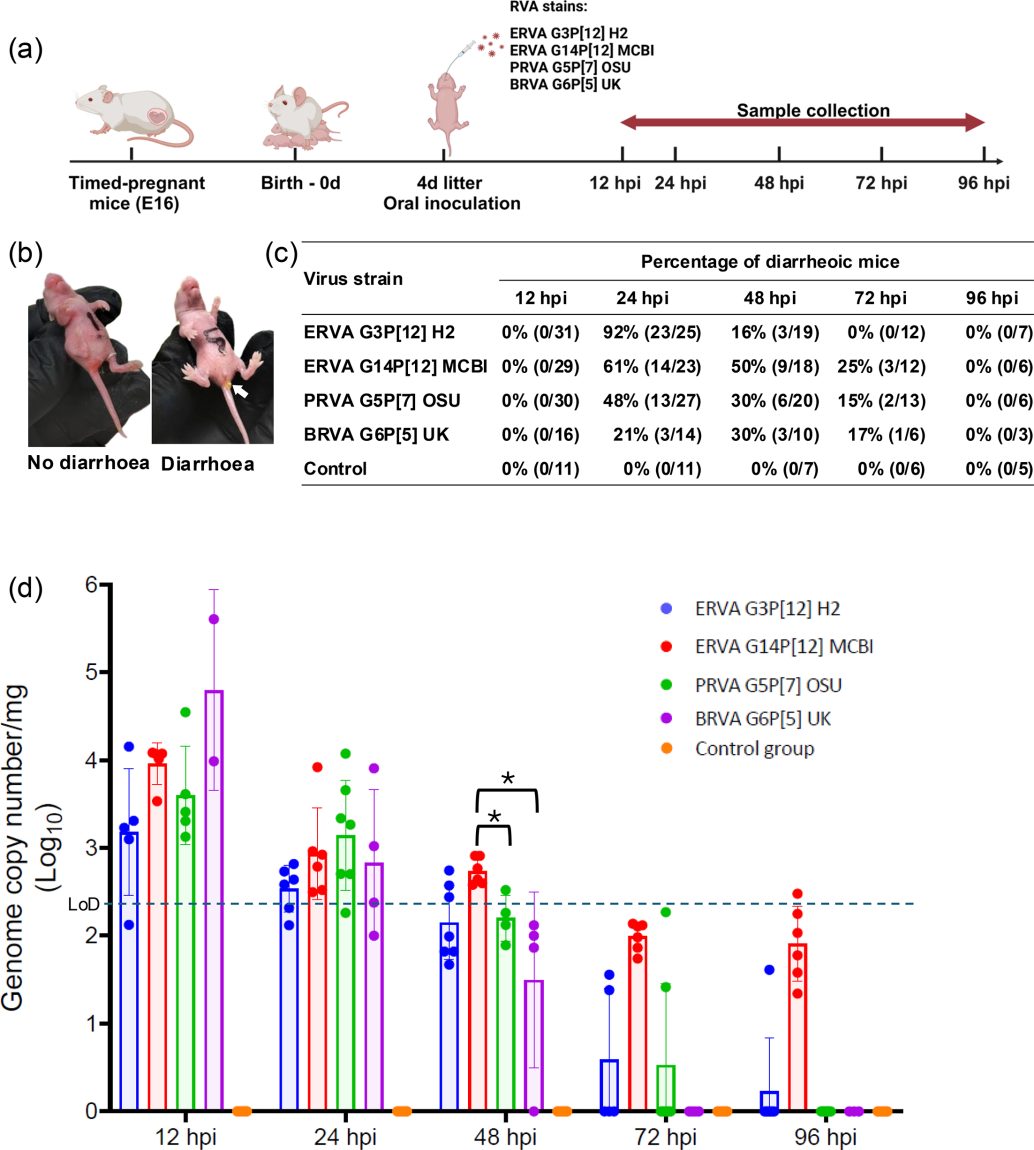
Experimental design, disease manifestation and viral load assessment following ERVA G3P[12], ERVA G14P[12], PRVA G5P[7] and BRVA G6P[5] infection in neonatal mice. (**a**) Overview of the experimental design. Four-day-old neonatal mice derived from timed-pregnant BALB/c mice (E17) were orally infected with 100 µl containing 1×10^7^ TCID_50_ of ERVA G3P[12], ERVA G14P[12], PRVA G5P[7] or BRVA G6P[5] and monitored for up to 96 hpi. Sampling timepoints included 12, 24, 48, 72 and 96 hpi. (**b**) Neonatal BALB/c mouse exhibiting yellowish loose diarrhoea, marked for identification. (**c**) A variable proportion of infected mice developed diarrhoea. (**d**) Viral genomic copy numbers per mg of intestine following infection. Statistical comparison of viral genome copy numbers per mg of intestine across RVA strains at multiple time points revealed significant differences (Kruskal–Wallis test, *P*<0.05). Only at 48 hpi, ERVA G14P[12] MCBI exhibited significantly higher viral genomic copy number per mg compared to PRVA G5P[7] OSU and BRVA G6P[5] UK (Dunn post-hoc test, *P values=0.0322 and 0.0294).

All timed-pregnant mice (*n*=23) gave birth on the 20th day of gestation (E20) from the first detection of an ejaculatory plug. On average, each pregnant mouse produced a litter of 6 (±1) pups. One timed-pregnant female was randomly allocated to each virus group (including the mock-infected group) and at each sampling timepoint [12, 24, 48, 72 and 96 h post-infection (hpi)]. At 4 days of age, pups were administered either a 100 µl dose containing 1×10^7^ TCID_50_ of each RVA strain in MEM containing 5% (w/v) sodium bicarbonate or an equal volume of MEM containing 5% (w/v) sodium bicarbonate (mock-infected group) via oral gavage using plastic feeding tubes (Cat# FTP-22-25-50, Instech Laboratories, Inc., Plymouth Meeting PA, USA) (Fig. 1a). Mice were returned to dams and monitored for 1 h at 15 min intervals following inoculation for any respiratory distress due to possible aspiration. No mortality was observed following inoculation, and all pups survived until their scheduled euthanasia time points.

### Clinical monitoring and sample collection

Mice were monitored at each specific time point for diarrhoea, evident as perianal staining or following gentle palpation of the abdomen (Fig. 1b). Randomly selected pups from the dam were randomly assigned to each time point and euthanized at 12, 24, 48, 72 and 96 hpi following infection with each RVA strain. The gastrointestinal tract was dissected, and a 5 cm segment of small intestine consisting of 1 cm of the aboral portion of the duodenum and 4 cm of jejunum was collected in a sterile microcentrifuge tube and stored at −80 °C until nucleic acid extraction was performed. The remaining intestinal tract was arranged as a Swiss roll on a wooden plate and fixed in a 10% neutral buffered formalin solution for 24 h.

### Sample processing, nucleic acid isolation and determination of viral genomic copies by reverse transcription quantitative PCR

A calibrated analytical balance was used to determine the weight of a 5 cm segment of small intestine, which was collected immediately after dissection. Tissue homogenates were then prepared at a 10% (w/v) concentration using sterile PBS (e.g. 100 mg tissue in 1,000 µl 1X PBS) and homogenized under sterile conditions using a Bead Ruptor Elite machine (Cat# 19-042E, Omni International, Kennesaw, GA, USA) with sterile PBS (1X; pH=7.4) using two cycles of 30 s at a speed of 4.0 m s^−1^. Nucleic acid isolation was performed using the taco^™^ mini Nucleic Acid Automatic Extraction System (GeneReach USA, Lexington, MA, USA) as previously described [39]. Two hundred microlitres of 10% tissue homogenate were extracted and eluted in equal volume of elution buffer. Extracted nucleic acids were stored at −80 °C until used. To quantify viral genomic copy numbers, an NSP3-specific reverse transcription quantitative PCR (RT-qPCR) (pan-rotavirus A, targeting the NSP3 gene) assay was performed as previously described [39]. The primers NVP3-FDeg (ACCATCTWCACRTRACCCTC) and NVP3-R1 (GGTCACATAACGCCCCTATA) and the probe NVP3-Probe (Cy5-ATGAGCACAATAGTTAAAAGCTAACACTGTCAA-BHQ2) were used. The reaction was prepared using the QuantiTect^™^ Probe RT-PCR kit (Qiagen, Hilden, Germany) in a 25 µl total volume, including 12.5 µl of 2X QuantiTect^™^ Probe RT-PCR Master Mix with ROX, 0.25 µl QuantiTect^™^ RT Mix, 1.25 ul of 20× Primer-Probe Mix [4 µl of forward and reverse primers and 4 µl of the probe (200 nM TaqMan^®^ fluorogenic probe) in 88 µl of Tris-EDTA buffer] and 5 µl of template RNA, which had undergone an initial denaturation step at 95 °C for 5 min. Reverse transcription and amplification were performed on an ABI 7500 Fast Real-Time PCR System (Applied Biosystems^®^, Life Technologies, Grand Island, NY, USA) with the following thermal cycling conditions: an initial reverse transcription step at 50 °C for 20 min, an activation step at 95 °C for 15 min, followed by 40 cycles of denaturation (94 °C for 45 s) and annealing/extension (60 °C for 90 s). A standard curve was generated using *in vitro*-transcribed RNA as previously described, spanning concentrations from 10⁷ to 10¹ RNA copies μl^−1^, and the analytical limit of detection (LoD) was determined as 270 genomic copies mg^−1^ [39].

### Histopathology

Following fixation in 10% neutral buffered formalin for 24 h, intestinal samples were processed routinely and embedded in paraffin. Four-micron sections of formalin-fixed, paraffin-embedded (FFPE) intestinal tissue were prepared and stained with haematoxylin and eosin (H and E) using standard procedures for histopathological analysis. Sections were made blindly and examined under a light microscope by two veterinary pathologists (WH and MC). Histological changes were scored based on a modified semi-quantitative scheme [40] assessing alterations in the villi, degree of oedema and congestion within the lamina propria, enterocyte vacuolation, neutrophilic infiltration, crypt hyperplasia and villous blunting (Table 1).

**Table 1.** Scoring system used for assessing intestinal histological alterations in ERVA, PRVA and BRVA-infected neonatal mice

Histological parameter	Criteria	Score
Villus scalloping	None	0
	Mild	1
	Moderate	2
	Marked	3
Lamina propria alterations	None	0
	Mild to moderate oedema/congestion	1
	Marked oedema/congestion	2
Enterocyte vacuolation	None	0
	Mild	1
	Moderate	2
	Marked	3
Presence of neutrophils	Within normal limits	0
	Increased numbers	1
Intraluminal cell debris	Absent	0
	Present	1
Crypt hyperplasia	None	0
	Mild to moderate	1
	Marked	2
Maximum score		12

### RNAscope^®^ *in situ* hybridization assay for detection of ERVA, PRVA and BRVA RNA

For RNAscope^®^ *in situ* hybridization (ISH), four anti-sense probes (Table 2) specifically targeting genomic segment 6 (encoding for VP6) were designed and synthesized by Advanced Cell Diagnostics (ACD, Newark, CA, USA). The RNAscope^®^ ISH assay was performed using the RNAscope 2.5 LSx RED Reagent Kit (ACD) on the automated BOND RXm platform (Leica Biosystems, Buffalo Grove, IL) as described previously [41]. Four-micron sections of FFPE tissues were mounted on positively charged Superfrost^®^ Plus Slides (VWR, Radnor, PA, USA) and subjected to automated baking and deparaffinization, followed by heat-induced epitope retrieval using a ready-to-use EDTA-based solution (pH 9.0; Leica Biosystems) at 100 °C for 15 min. Subsequently, tissue sections were treated with a ready-to-use protease (RNAscope^®^ 2.5 LSx Protease, ACD) for 15 min at 40 °C, followed by a ready-to-use hydrogen peroxide solution for 10 min at room temperature. Slides were then incubated with the ready-to-use probe mixture for 2 h at 40 °C, and the signal was amplified using a specific set of amplifiers (AMP1 through AMP6 as recommended by the manufacturer). The signal was detected using a Fast Red solution for 10 min at room temperature. Finally, slides were counterstained with a ready-to-use haematoxylin for 5 min, followed by five washes with 1X BOND Wash Solution (Leica Biosystems). Slides were rinsed in deionized water, dried in a 60 °C oven for 30 min and mounted with Ecomount^®^ (Biocare, Concord, CA, USA).

**Table 2.** Segment 6 (VP6) specific probes developed for RNAscope^®^ ISH

Rotavirus strain	Target region (bp)	GenBank accession no.
ERVA G3P[12] H2	7–1,321	KC815684.1
ERVA G14P[12] MCBI	65–1,177	KJ628067.1
BRVA G6P[5] UK	2–1,097	KC215501.1
PRVA G5P[7] OSU	89–1,174	KR052760.1

### Statistical analysis

Statistical analyses were performed using GraphPad Prism 9 (GraphPad Software, San Diego, CA, USA) and JMP Pro 18 (JMP Statistical Discovery LLC, Cary, NC, USA). Diarrhoea incidence data were analysed using a generalized linear mixed model with a binomial distribution in JMP Pro 18. Virus strain was included as a fixed effect, and dam was specified as a random effect to account for clustering of pups within litters. The mean of histology scores and viral genome copy numbers for each virus strain was calculated, and the Kruskal–Wallis non-parametric rank sum test was used to compare groups. Differences in viral genome copy number per mg of intestine across time points and RVA strains were analysed using JMP Pro 18. A Kruskal–Wallis rank sum test was used for overall group comparisons, followed by non-parametric pairwise comparisons using the Dunn method for joint ranking. *P* values were adjusted for multiple comparisons using the Bonferroni correction. The relationship between the proportion of mice with diarrhoea at the time of sacrifice and their respective histology scores was assessed using Spearman’s rank correlation coefficient. The level of significance was set at *P*<0.05 for all tests.

## Results

### Neonatal mice are susceptible and develop diarrhoea following oral infection with ERVA G3P[12] and G14P[12], PRVA G5P[7] and BRVA G6P[5]

Following oral inoculation of suckling BALB/c mice with 1×10^7^ TCID_50_/100 µl of the four RVA strains (ERVA G3P[12] H2, ERVA G14P[12] MCBI, PRVA G5P[7] OSU and BRVA G6P[5] UK), diarrhoea, characterized by loose yellow faeces (Fig. 1b), was observed. No diarrhoea was identified in the mock-infected control groups (Fig. 1b). For all RVA strains used in this study, diarrhoea was first identified at 24 hpi (Fig. 1c). For ERVA G3P[12] H2 strain, the incidence of diarrhoea peaked at 24 hpi with 92% of mice affected, but this rapidly declined to 16% at 48 hpi and 0% at subsequent time points (Fig. 1c). While the ERVA G14P[12] MCBI strain also induced diarrhoea with a peak at 24 hpi comprising 61% of the inoculated mice, 50% of the animals exhibited diarrhoea at 48 hpi, decreasing to 25% at 72 hpi and resolving entirely by 96 hpi (Fig. 1c). Similarly, infection with the PRVA G5P[7] OSU strain led to a peak in diarrhoea at 24 hpi with 48% of mice affected, which decreased to 30% at 48 hpi and further to 15% by 72 hpi, with all cases resolved by 96 hpi. The BRVA G6P[5] UK strain also induced diarrhoea at 24 hpi with 21% of infected mice, peaked at 48 h (30%), then decreased to 17% at 72 hpi and resolved at 96 hpi. Even though ERVA G3P[12] H2 induced a higher rate of diarrhoea at 24 hpi compared to other RVA strains, no statistically significant differences in the incidence of diarrhoea across strains were identified (*P* value>0.05). Finally, no mortalities were recorded for any of the RVA strains used. Overall, the ERVA strains induced the highest proportion of diarrhoea compared to the PRVA and BRVA strains.

### Limited rotaviral intestinal replication among ERVA, PRVA and BRVA strains in neonatal mice

Viral RNA was detectable in the small intestine of all four infected groups starting at 12 hpi, with mean viral genomic copy numbers per mg of intestine ranging between (1.6±0.5)×10³ and (6.3±10.0)×10⁴ copies mg^−1^ (Fig. 1d). Viral genomic copy numbers decrease gradually over time. By 24 hpi, viral genomic copy numbers per mg had a 10-fold reduction, with mean values between (3.2±0.2)×10² and (1.3±0.4)×10³ copies mg^−1^. By 72–96 hpi, viral loads were mostly below the LoD for ERVA G3P[12] H2, ERVA G14P[12] MCBI, PRVA G5P[7] OSU and BRVA G6P[5] UK (Fig. 1d). Interestingly, viral RNA was still detected at 96 hpi for ERVA G14P[12] MCBI and ERVA G3P[12] H2 at 79±25 and 1.6±4.0 genome copy numbers per mg, respectively. Statistical comparisons of viral genome copy numbers per mg of intestine at multiple time points among RVA strains revealed only a significantly higher number for ERVA G14P[12] MCBI compared to both PRVA G5P[7] OSU and BRVA G6P[5] UK at 48 hpi (*P* values=0.0322 and 0.0294, respectively). No other statistically significant differences were identified. Overall, RVA replication was limited with similar dynamics across ERVA, PRVA and BRVA strains used, with values below the LoD by 72 hpi in all cases.

### Equine, porcine and bovine RVA induce intestinal histological lesions that correlate with clinical disease and viral tropism

**Fig. 2.**
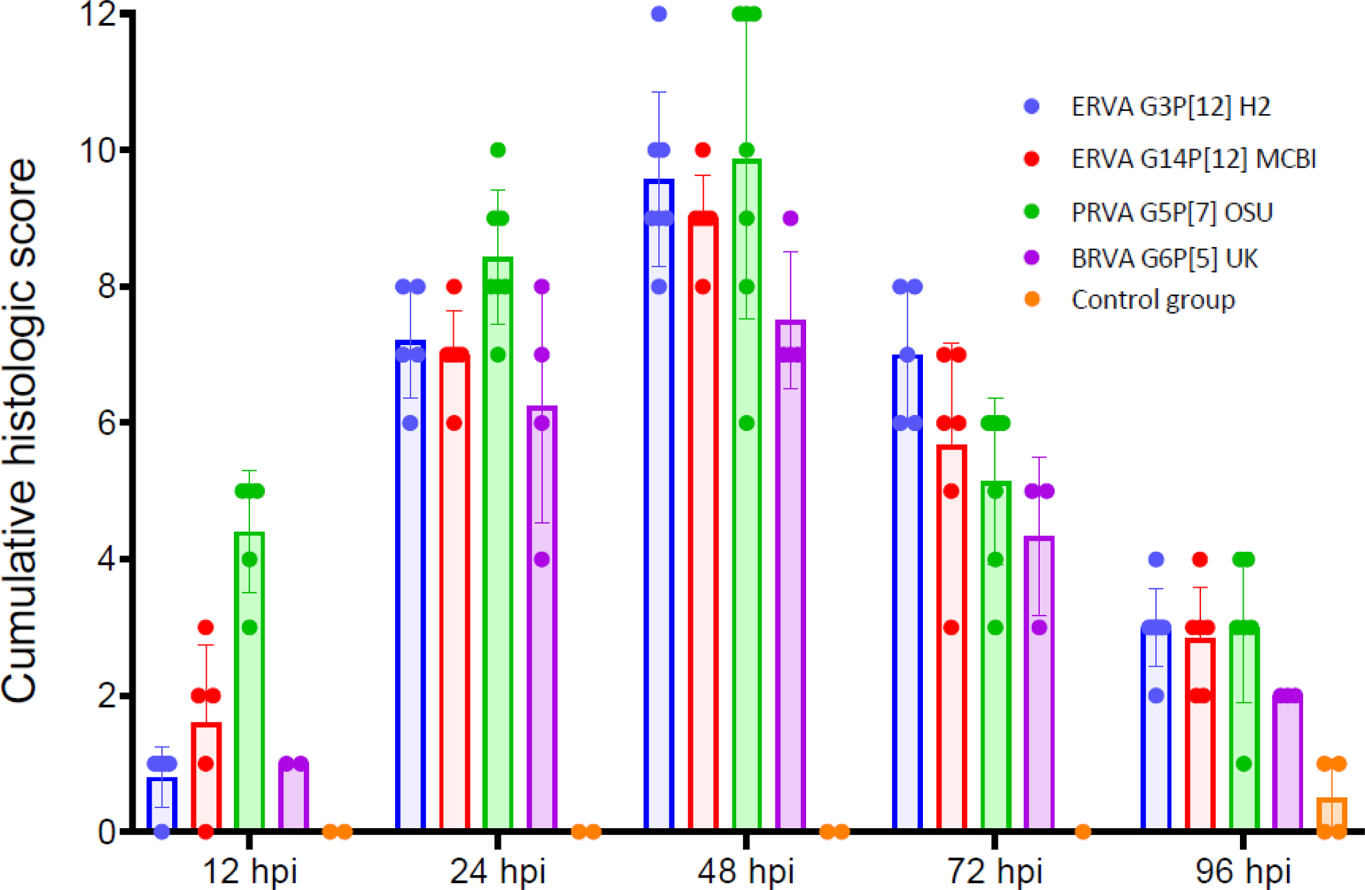
Temporal dynamics of intestinal lesions following infection of neonatal mice with ERVA G3P[12], ERVA G14P[12], PRVA G5P[7] and BRVA G6P[5]. Cumulative scores were determined following analysis of six histological parameters (villus scalloping, lamina propria alterations, enterocyte vacuolation, presence of neutrophils, intraluminal cell debris and crypt hyperplasia). Mock-infected mice (control group) are depicted in orange. Histological alterations reached their maximum severity at 48 hpi with no significant differences between RVA strains across experimental timepoints (*P*>0.05). Histological lesions declined by 96 hpi but did not return to baseline values. Each bar represents the mean for lesion severity at a given time point and error bars indicating sd.

**Fig. 3.**
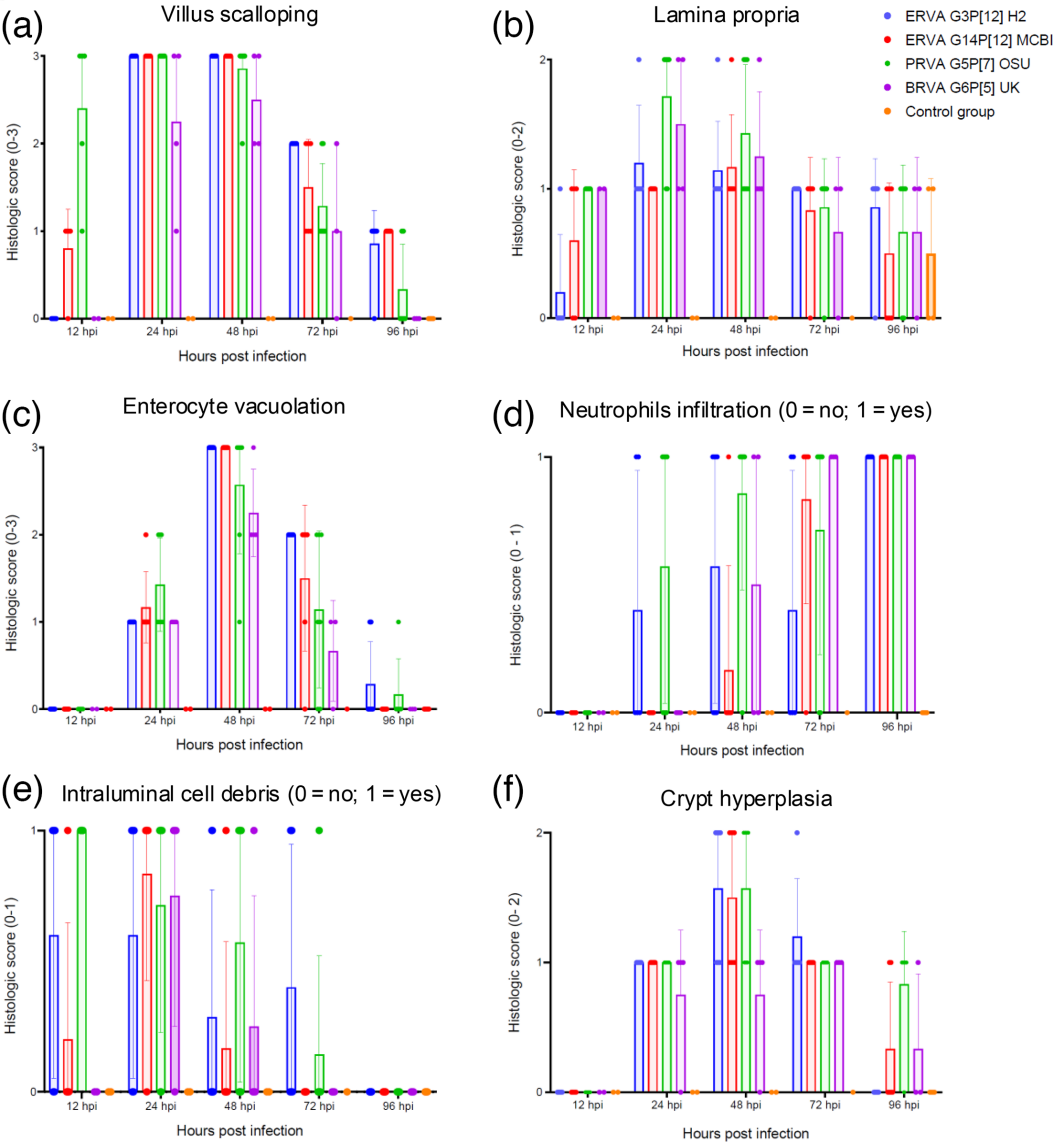
Temporal progression of individual histological intestinal parameters evaluated following infection of neonatal mice with ERVA G3P[12], ERVA G14P[12], PRVA G5P[7] and BRVA G6P[5]. Panels show lesion severity or presence/absence for six distinct histological parameters: (**a**) villus scalloping, (**b**) lamina propria integrity, (**c**) enterocyte vacuolation, (**d**) neutrophil infiltration (0=no; 1=yes), (**e**) intraluminal cell debris (0=no; 1=yes) and (**f**) crypt hyperplasia. Each bar represents the mean of the parameter, and the error bar indicates the sd.

**Fig. 4.**
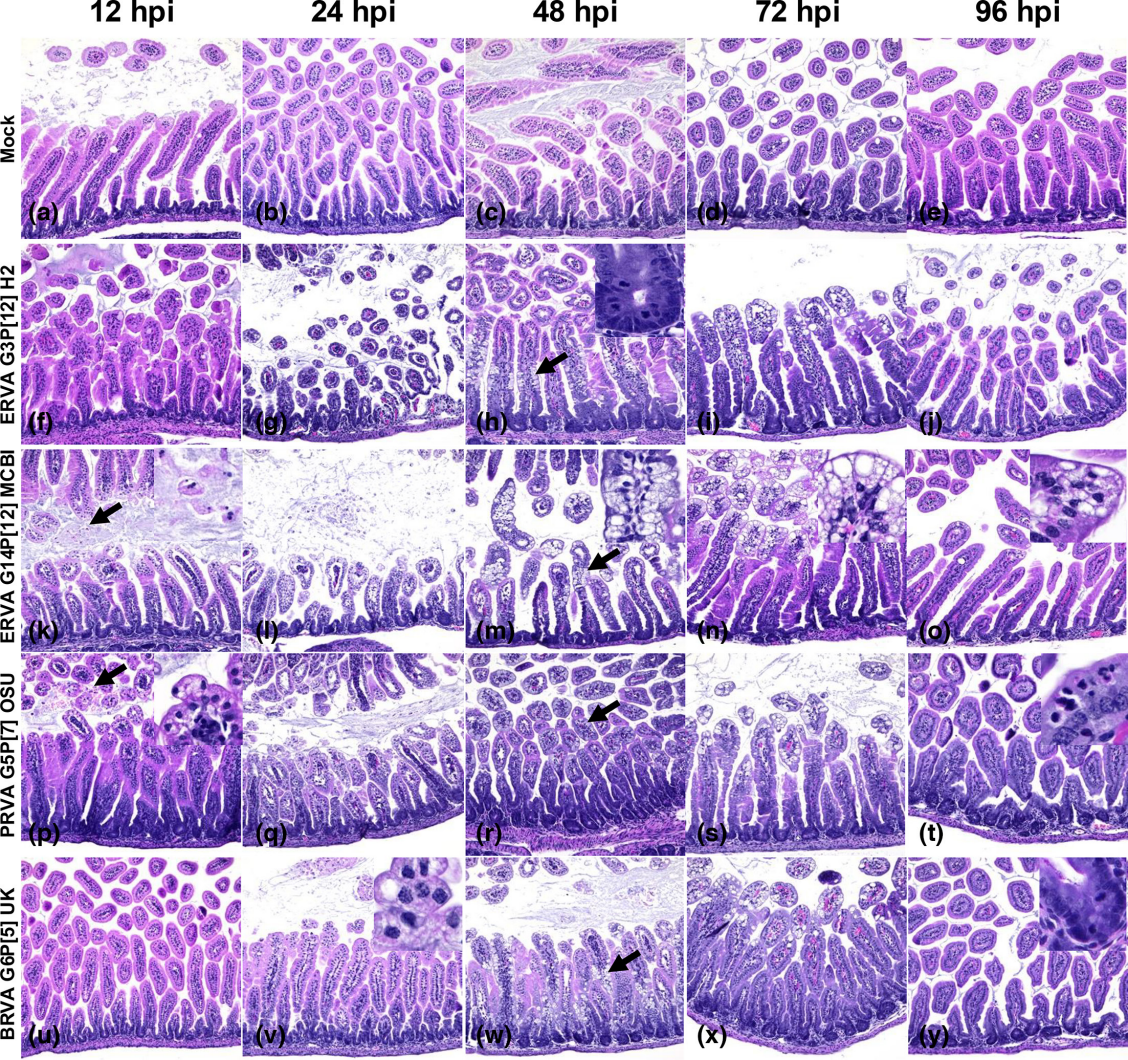
Histological lesions in the small intestine of infected neonatal mice. Image panel depicts (**a–e**) mock-infected, (**f–j**) ERVA G3P[12]-infected, (**k–o**) ERVA G14P[12]-infected, (**p–t**) PRVA G5P[7]-infected and (u–y) BRVA G6P[5]-infected neonatal mice at 12, 24, 48, 72 and 96 hpi. Changes at 12 hpi included scalloping [e.g. p (inset)] and intraluminal cell debris [e.g. k (arrow and inset)] that evolved to villus blunting (e.g. l) and enterocyte vacuolation [e.g. l, q, v (inset)] by 24 hpi. Prominent vacuolation was evident at 48 and 72 hpi [h, i, m, n, r, s, w, x (arrows)] as well as crypt hyperplasia (h, inset). At 96 hpi, crypt hyperplasia remains evident (e.g. **t and y**) with sporadic enterocyte vacuolation and transmigrating neutrophils (t, inset). H and E. Magnification 200X.

**Fig. 5.**
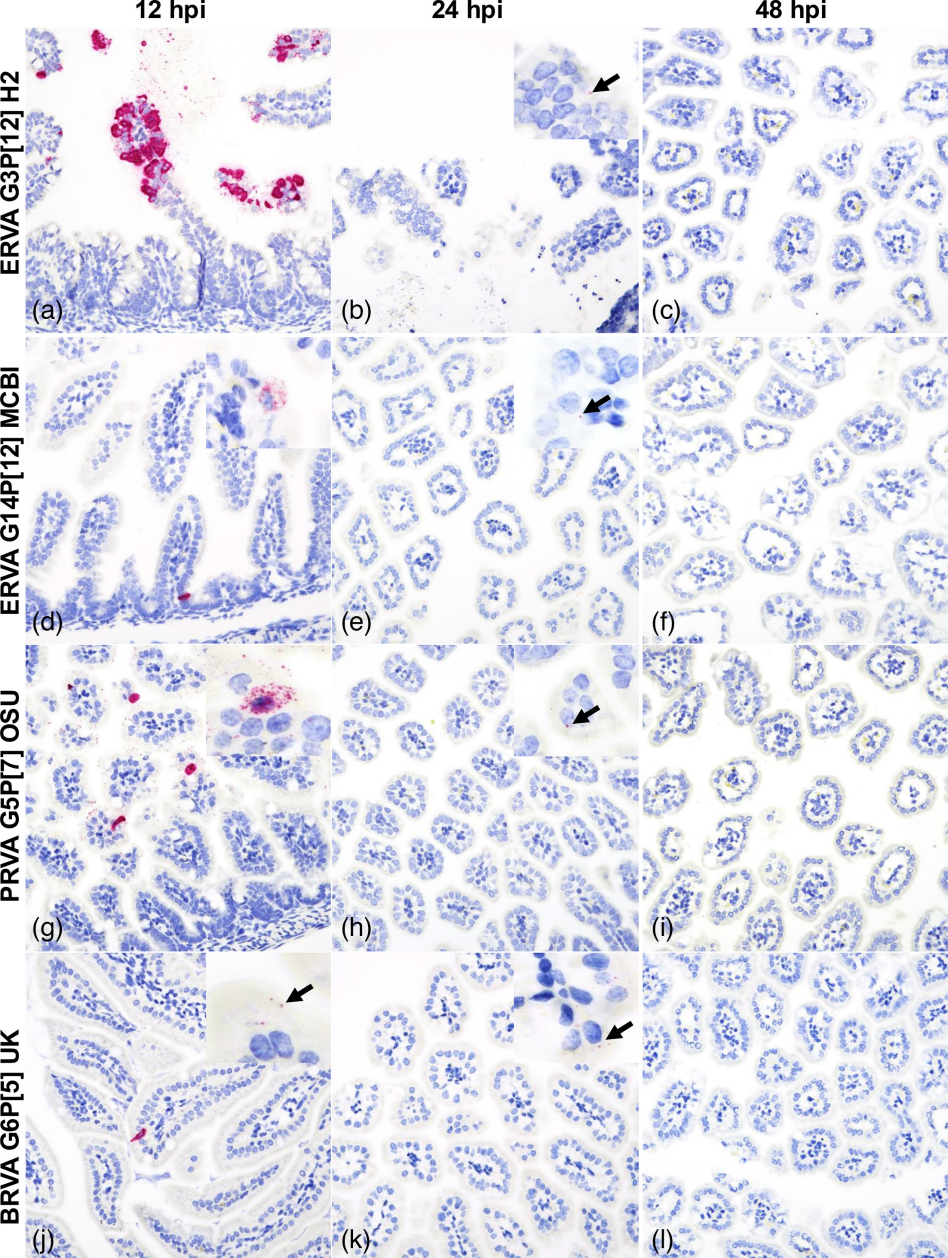
*In situ* detection of rotavirus RNA at 12, 24 and 48 hpi in the small intestine following infection of neonatal mice with ERVA G3P[12] H2, ERVA G14P[12] MCBI, PRVA G5P[7] OSU and BRVA G6P[5] UK. The bright red punctate to confluent staining represents hybridized viral RNA within infected epithelial cells (black arrows). (**a), (d), (g) and (j**) At 12 hpi, robust ISH signals were visible in enterocytes, indicating early viral replication. (**b), (e), (h) and (k**) By 24 hpi, there is a pronounced reduction in the number of infected enterocytes and in the abundance of the RNA signal. (**c), (f), (i) and (l**) By 48 hpi, viral RNA was undetectable by ISH, suggesting viral clearance. Magnification 400X.

The histological alterations across rotavirus strains ERVA G3P[12] H2, ERVA G14P[12] MCBI, PRVA G5P[7] OSU and BRVA G6P[5] UK were similar, with early alterations starting as early as 12 hpi and extending up to 96 hpi despite viral clearance in most cases (Figs2^4^). The histological alterations were overall characterized by the parameters indicated in Table 1 and depicted in Figs3  4. At 12 hpi, ERVA G14P[12] MCBI and PRVA G5P[7] OSU had the most pronounced alterations at this earliest timepoint (1.6±1.1 and 4.4±0.9, respectively), while ERVA G3P[12] H2 and BRVA G6P[5] UK showed milder effects (0.8±0.5 and 1.0±0.0, respectively). The intestinal mucosa showed mild congestion and oedema for all strains, with variable amounts of intraluminal cell debris in all strains except BRVA G6P[5] UK (e.g. Fig. 4k, inset). In mice infected with ERVA G14P[12] MCBI and PRVA G5P[7] OSU, the intestinal villi showed early evidence of scalloping of apical enterocytes, most prominent in mice infected with PRVA (Fig. 4p). Correlating with these early histological alterations, cytoplasmic rotaviral RNA was detectable via RNAscope^®^ ISH within villus enterocytes across all RVA strains (Fig. 5a, d, g and j).

By 24 hpi, histological alterations increased in severity across all RVA strains, with a higher degree of mucosal congestion and oedema, mild to moderate enterocyte cytoplasmic vacuolation, mild crypt hyperplasia, intraluminal cell debris and villus scalloping (scores of 7.2±0.8, 7.0±0.6, 8.4±1.0 and 6.3±1.7 for ERVA G3P[12] H2, ERVA G14P[12] MCBI, PRVA G5P[7] OSU and BRVA G6P[5], respectively), the latter being less prominent in BRVA G6P[5]-infected mice (Figs2^4^). Scattered to rare enterocytes showed cytoplasmic rotaviral RNA labelling via RNAscope^®^ ISH (Fig. 5b, e, h and k).

At 48 hpi, the peak score of histological alterations was reached across all strains, with all changes being moderate and predominantly characterized by pronounced cytoplasmic vacuolation of enterocytes typically within the apical two-thirds of the intestinal villi (Fig. 4h, m, r and w) and decreasing amounts of intraluminal cell debris (Figs2  3). Mean scores ranged between 7.5 and 9.9 (sd ranging from 0.6 to 2.3), respectively, with the most severe histological changes noted in the ERVA G3P[12] and PRVA G5P[7]-infected mice and the least severe in the BRVA G6P[5]-infected mice. Intestinal crypt hyperplasia became one of the most prominent changes in infected mice except for BRVA G6P[5]-infected mice (Fig. 4h inset). No rotaviral RNA was detected within enterocytes via RNAscope^®^ ISH at this time point (Fig. 5c, f, i and l).

At 72 hpi, histological alterations start to decrease in severity, with apical villi scalloping and enterocyte cytoplasmic vacuolation predominantly confined to the apical one-third of the intestinal villi (Figs2  3). Intestinal crypt hyperplasia remained prominent. The ERVA G3P[12] H2-infected mice had the highest overall score (7.0±1.0), while BRVA G6P[5] UK-infected mice had the lowest (4.3±1.2) (Fig. 2).

By 96 hpi, scores decreased similarly across all RVA strains (Figs2  4). At this time point, intestinal crypt hyperplasia decreases in severity across all strains, returning to baseline in the ERVA G3P[12] H2-infected group (Fig. 3) . Interestingly, increased mucosal neutrophils that transmigrate through the enterocyte lining are noted in the PRVA G5P[7] OSU-infected animals (Fig. 4t).

While no statistically significant differences in terms of histological scores were identified between RVA strains across experimental time points (*P* values>0.05), we determined a positive correlation between the occurrence of diarrhoea and the development of histological intestinal alterations (Table 3) for all strains but the ERVA G3P[12] H2 strain, for which the correlation was determined to be very weak and not statistically significant (Spearman’s *r*=0.1809, *P*=0.398). This is likely attributed to the rapid resolution of diarrhoea in this group, with a 76% reduction in diarrhoeic mice within a 12 h window between 24 and 48 hpi (Fig. 1b). Overall, we have demonstrated that the development of diarrhoea is associated with histological alterations in the small intestine and early viral tropism for intestinal enterocytes that show similar dynamics across all RVA strains except for ERVA G3P[12], in which a slower onset of histological lesions was noted.

**Table 3.** Correlation between histological intestinal scores and incidence of diarrhoea following infection of neonatal mice with ERVA, PRVA and BRVA strains

Correlation statistic	ERVA G3P[12] H2	ERVA G14P[12] MCBI	PRVA G5P[7] OSU	BRVA G6P[5] UK
Spearman correlation coefficient	0.18	0.65	0.81	0.64
95% CI	−0.252 to 0.553	0.364 to 0.825	0.642 to 0.907	0.204 to 0.868
*P* value (two-tailed)	0.398	<0.001	<0.001	0.009

CI, Confidence Interval.

## Discussion

While the neonatal (suckling) mouse model has been employed to investigate RVA pathogenesis and evaluate vaccine efficacy [42,44], a significant gap exists in establishing a well-characterized mouse model that could be utilized explicitly for ERVA pathogenesis and vaccinology research. Even though a recent study has reported the use of a similar model to assess vaccine efficacy [45]. Data reported from this model only included the development of diarrhoea without further assessment of other parameters during the experimental challenge. This study addresses that gap, offering a foundation for future research in vaccine development and pathogenesis studies related to ERVA. Additionally, even though previous studies have investigated maternal antibody transfer [46,49], T-cell-mediated immunity [50,52], and protective responses to vaccines, and studies on host range restriction [53,56], none thoroughly characterized the time course-based replication dynamics, histopathological changes and viral tropism following rotavirus infection in neonatal mice.

Here, we comparatively and methodically evaluated the infection dynamics of ERVA, PRVA and BRVA strains in neonatal mice, demonstrating for the first time that ERVA G3P[12] and G14P[12] strains are capable of infecting suckling mice similarly to PRVA and BRVA strains. Infected animals developed transient diarrhoea and histological alterations that temporally correlated with clinical disease. Moreover, these alterations were associated with enterocyte infection during the initial 24 hpi, after which both viral load and the number of infected enterocytes declined. Thus, despite using these strains on a non-natural host, neonatal mice develop both a clinical and pathological disease phenotype that is useful to understand certain aspects of ERVA pathogenesis and to assess vaccine efficacy. Our comparative analysis across a spectrum of RVA strains demonstrated similar infection dynamics, with peak viral loads occurring at 12–24 hpi, declining below detectable levels by 72–96 hpi, whereas earlier studies conducted in neonatal mice infected with the murine RVA wild-type strain epidemic diarrhoea of infant mice reported prolonged viral shedding [57,58]. In contrast, histopathological changes in our study, including enterocyte vacuolation, scalloping and crypt hyperplasia, peaked at 48 hpi and persisted through at least 96 hpi.

The host range restriction among RVA strains is a significant feature recognized as a limiting factor for effective interspecies transmission, although such transmission events continue to occur in nature [54,59]. In our study, viral replication dynamics do not follow the expected course that they would in a highly susceptible (natural) host but instead sharply decline in the subsequent 12 h intervals following inoculation evaluated in this study. This reflects the host-level restriction for the RVA strains used in neonatal mice, indicating poor adaptation and limited replication in non-host species. While this is not an ideal outcome for a challenge model (and represents a limiting factor of this model) since the determination of viral outputs is an important parameter to consider in efficacy studies of vaccines and therapeutics, histological alterations were directly related to virus cytopathogenicity to the enterocyte lining, and, therefore, lesion development and histopathological evaluation are still important phenotypes to be monitored in challenge/efficacy studies.

While not statistically different compared to other RVA strains, the ERVA G3P[12] H2 strain induced a higher proportion of diarrhoea at 24 hpi. We can only hypothesize that this difference could be related to the amino acid composition at the receptor binding domain of VP8*. Our previous study comparing the ERVA G3P[12] H2 strain with the ERVA G14P[12] MCBI strain demonstrated 12 amino acid substitutions in VP8*, with 9 located in the variable region and haemagglutination domain of VP8* [17]. We therefore hypothesize that the VP4 protein of the ERVA G3P[12] H2 strain may exhibit greater affinity for cellular receptors in murine enterocytes compared to that of the ERVA G14P[12] MCBI strain.

Our study agrees with previous findings on rotavirus-induced intestinal pathology in suckling mice, confirming early enterocyte vacuolation, villus atrophy and crypt hyperplasia as key histopathological features [42]. However, previous studies assessing histological alterations in the murine intestine following RVA infection have only identified enterocyte vacuolation as the most significant intestinal change [42,60,62]. We have performed a detailed microscopic evaluation and identified several other cellular and tissue alterations in addition to cellular vacuolation that are associated with RVA infection and that serve as relevant parameters for comprehensive scoring of intestinal alterations, such as villus scalloping (an early cellular change) and crypt hyperplasia (an indication of a reparative response). While villus blunting was appreciated in a few infected animals, this was not a consistent histological alteration as known to be the case in animals infected with host-specific RVA strains. Interestingly, ERVA, PRVA and BRVA strains used in this study induced comparable microscopic changes with similar dynamics. In all cases, these changes persisted, albeit with low severity, even after clearance of RVA-infected enterocytes at 48 hpi and beyond. Some strains, such as PRVA G5P[7] OSU, still exhibited focal transmigration of neutrophils at 96 hpi, highlighting that intestinal lesions do not resolve concurrently with that of clinical disease.

Unlike previous studies, our study integrates histological parameters, viral load analysis and RNAscope^®^ ISH to offer a more comprehensive assessment of heterologous RVA infections in neonatal mice. Our findings reinforce the utility of the neonatal mouse model in studying heterologous RVA infections, although with some limitations. Even though viral replication is limited, ERVA G3P[12] and G14P[12] successfully established enterocyte infection as previously established for PRVA and BRVA, inducing diarrhoea correlated with pronounced intestinal lesions. Hence, the development of this disease phenotype characterized by diarrhoea and histological intestinal alterations would still be considered relevant for assessing the preclinical efficacy of vaccines and other interventions against ERVA or other RVA.

This study has several limitations. While neonatal mice offer a convenient and reproducible surrogate model, species-specific host restriction limited intestinal viral replication, emphasizing the fact that this animal model is not perfect, and many aspects related to rotavirus biology can only be addressed using the natural host. However, it should be acknowledged that experimental infection models in foals are logistically challenging compared to other livestock species. While not the focus of this study per se, we have not assessed adaptive immune responses following infection of suckling mice based on the rationale that new vaccines for horses and other livestock would still likely rely on vaccination of pregnant dams to enhance maternal immunity as RVA infections in livestock can occur within the first few hours following parturition. It is important to also consider that key anatomical and immunological differences between neonatal mice and foals may influence RVA infection outcomes. Mice have a shorter gastrointestinal tract, distinct enterocyte glycosylation and faster epithelial turnover, which can limit viral replication [63]. Additionally, their immature immune system, characterized by delayed mucosal IgA responses and Th2 bias, contrasts with the more developed gastrointestinal-associated lymphoid tissue and immune responses in neonatal foals [64,65]. These differences should be considered when interpreting mouse model data. Future studies focusing on neonate adaptive responses are, therefore, warranted. Finally, we did not investigate the effects of rotavirus infection on the microbiota or predisposition to secondary bacterial infections. Experiments were performed in specific pathogen-free facilities, and there was no evidence of secondary bacterial enteritis when examined microscopically. Additional studies focusing on this topic are needed to shed light on the interaction between RVA and opportunistic microbiota.

## Conclusion

In the present study, neonatal mice demonstrated a transient disease phenotype characterized by diarrhoea and pronounced histological intestinal alterations following infection with equine, porcine and bovine RVA strains. While the restricted viral replication observed in the small intestine likely indicates host-specific limitations, there is still significant potential for using this mouse model at the preclinical stage for evaluating novel vaccines and therapeutics, thus providing a potential preclinical model.

## Abbreviations

ACD: Advanced Cell Diagnostics
BRVA: bovine rotavirus A
ERVA: equine rotavirus A
FFPE: formalin-fixed, paraffin embedded
H and E: haematoxylin and eosin
hpi: h post-infection
ISH: *in situ* hybridization
LoD: limit of detection
MEM: minimum essential medium
PRVA: porcine rotavirus A
RT-qPCR: reverse transcription quantitative PCR
RVA: group A rotavirus
TCID₅₀: tissue culture infectious dose 50%
